# Thermodynamics of the Micellization of a Simple Zwitterionic Surfactant in the Presence of Imidazolium Salts

**DOI:** 10.1021/acsomega.5c08858

**Published:** 2025-11-04

**Authors:** Álvaro Javier Patiño-Agudelo, Nicolas Keppeler, Lucas Mendel de Oliveira Silva Martins, Frank H. Quina

**Affiliations:** † Departamento de Química Fundamental, Instituto de Química, 28133 Universidade de São Paulo, 05508-000 São Paulo, SP Brazil; ‡ Departamento de Físico-Química, Instituto de Química, Universidade Estadual de Campinas, 13083-970 Campinas, SP Brazil

## Abstract

Although formally neutral, in aqueous media, zwitterionic micelles selectively concentrate the anions of added inorganic salts at the micelle-water interface. The resultant negative electrostatic potential of the micelles can be additionally modulated by the metal cations of the added salt. In the present work, isothermal titration calorimetry (ITC) has been employed to determine the critical micelle concentrations (CMC) and standard thermodynamic parameters of micellization (Δ*G*_mic_^0^, Δ*H*_mic_^0^, and Δ*S*_mic_^0^) of the zwitterionic surfactant *N*-tetradecyl-dimethylammoniumpropanesulfonate (SB3–14) in aqueous solution at 298.15 K as a function of the concentration (100–1000 mmol L^–1^) of six salts with organic cations: the chloride salts of 1-methylimidazolium (C_o_mimCl) and 1-ethyl-3-methylimidazolium (C_2_mimCl) and the chloride, bromide, iodide, and perchlorate salts of 1-butyl-3-methylimidazolium (C_4_mimCl, C_4_mimBr, C_4_mimI, and C_4_mimClO_4_, respectively). C_o_mimCl, C_2_mimCl, C_4_mimCl, and C_4_mimBr increase the CMC of SB3–14 in this concentration range, while C_4_mimI and C_4_mimClO_4_ initially decrease the CMC, followed by an increase in the CMC at higher salt concentrations. Although the Δ*H*_mic_^0^ values of SB3–14 are consistently negative and the Δ*S*_mic_^0^ values decrease relative to those in the absence of added salt, the overall behavior is distinct from that in the presence of the corresponding sodium salts, especially for C_4_mimI and C_4_mimClO_4_ with the most strongly interacting anions. We demonstrate that, by explicitly incorporating specific cation-dependent interactions into the formalism developed earlier for inorganic salts [ Patiño-AgudeloÁ. J.,  Colloids Surf., A 2025, 725, 137728], it is possible to separate the contributions of the organic cation to the micellization of SB3–14 from those of the anion, providing a coherent picture of the complex interplay between anion, cation, and the zwitterionic interface.

## Graphical abstract

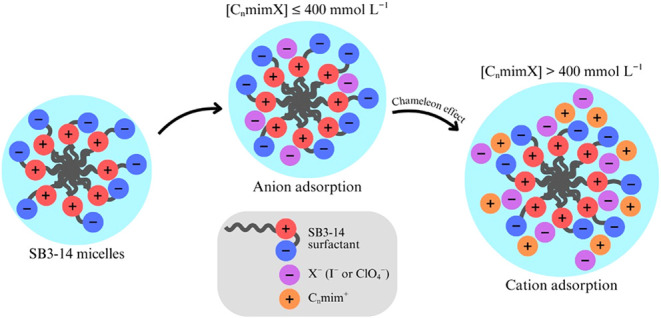

## Introduction

Zwitterionic surfactants are characterized by a hydrophobic portion coupled to a headgroup containing equal numbers of positive and negative charge centers, a simple example being the zwitterionic sulfobetaine surfactant SB3–14, which has a 14 carbon hydrophobic tail attached to a headgroup consisting of a dimethylammonium cationic center attached to a sulfonate anionic center by a three-carbon methylene segment: CH_3_(CH_2_)_13_-N­(CH_3_)_2_^+^(CH_2_)_3_-SO_3_^–^. Although formally neutral, in aqueous media, zwitterionic aggregates and monolayers exhibit the rather interesting property of preferentially and selectively concentrating or “binding” the anions of added salts, creating a net negative electrostatic potential at the interface.^1–6^ At higher added salt concentrations and degrees of association of anions with the micelle, cations of the salt are also attracted to the vicinity of the micelle, resulting in additional modulation of the net electrostatic potential of the micelles. In the case of purely inorganic salts, the effects of cations depend primarily on their valence, indicating that the interaction is largely electrostatic in nature.^7^ In contrast, the interactions of anions with the zwitterionic interface are markedly more specific. Large less-hydrated anions interact much more strongly with the interface than smaller, more highly hydrated anions, giving rise to a Hofmeister-like sequence of anion affinities for the interface of the type Cl^–^ < Br^–^ < I^–^ < ClO_4_^–^. Thus, in the presence of added salts, zwitterionic micelles are no longer uncharged, but rather have a “chameleon-like” nature due to the interplay between the interactions of the anions and cations of added salts with the micelle-water interface.^7,8^

A wide variety of techniques, both experimental and theoretical,^1,5,7,9–15^ have been employed to investigate the interactions between anions and zwitterionic micelles. Given the continuing interest in unraveling the origins and nature of the nonCoulombic contributions to the selectivity of ionic interactions with interfaces, we and others have recently employed isothermal titration calorimetry (ITC) to determine added salt effects on the standard enthalpies of the micellization of simple zwitterionic surfactants such as SB3–14 ^16^ or its 12-carbon homologue SB3–12 ^11^ and on the binding of anions to zwitterionic SB3–14 micelles above the critical micelle concentration (CMC).^17^ Interestingly, for a variety of sodium salts, the interaction of the anion with the zwitterionic interface was found to be enthalpically favorable but entropically unfavorable, with little or no influence of the sodium cation.

In the present work, we report analogous ITC measurements of the micellization of SB3–14 in the presence of salts with organic cations of increasing hydrophobicity derived from *N*-methylimidazole, *viz*., 1-methylimidazolium (C_0_mim^+^) and 1-ethyl- and 1-butyl-3-methylimidazolium (C_2_mim^+^ and C_4_mim^+^, respectively; refer to Scheme S1 of the Supporting Information file for the chemical structures). Alkylimidazolium salts are interesting in their own right as ionic liquids, but in the presence of water can aggregate and/or ion pair to form complex microheterogeneous solutions over a wide range of water mole fractions.^18–22^ Several previous studies^23–29^ have demonstrated that ionic liquid-based salts of this type affect the properties of zwitterionic micelles in ways that are, in several important aspects, distinct from those of the sodium salts employed in our previous studies. Thus, in addition to the specific anion-zwitterionic interfacial interactions, additional organic cation-zwitterionic interfacial interactions must be taken into consideration in interpreting the thermodynamic parameters of micellization. By explicitly incorporating cation-dependent interactions into the formalism developed earlier,^16^ we demonstrate that it is possible to separate the contributions of the cation to the enthalpy and entropy of micellization of SB3–14 from those of the anion, providing a coherent picture of the complex interplay between anion, cation, and the zwitterionic SB3–14 interface.

## Materials and Methods

### Materials

*N*-Methylimidazole (99%), *n*-butyl bromide (99%), *n*-butyl iodide (99%), ethyl bromide (98%), hydrochloric acid (37%), perchloric acid (70%), ethyl acetate (99%), and methanol (99.9%) were purchased from Sigma-Aldrich (St. Louis, MO, USA) and used without further purification. *N*-Tetradecyl-*N*,*N*-dimethyl-3-ammonio-1-propanesulfonate (SB3–14, Sigma-Aldrich 99%) was purified further by recrystallization from acetone-methanol.^30^ Deionized water (18 MΩ cm at 298.12 K) obtained from a Milli-Q II reverse osmosis system (Millipore, USA) was used to prepare all solutions.

#### Ionic Liquid Synthesis and Characterization

The imidazolium salts were prepared by the general procedures outlined in the work of Bioni et al.^31^

##### 1-Methylimidazolium Chloride (C_o_mimCl)

C_o_mimCl was prepared by neutralizing *N*-methylimidazole (0.1 mol) with aqueous HCl. Water was removed under reduced pressure by evaporation. The residue was washed with ethyl acetate and dried under a vacuum to yield the pure ionic liquid. ^1^H NMR (DMSO-*d*_6_, 300 MHz) δ 3.79 (s, 3H), 7.75 (s, 1H), 7.92 (s, 1H), 8.93 (s, 1H). Yield: 95%.

##### 1-Butyl-3-methylimidazolium Bromide (C_4_mimBr)

C_4_mimBr was synthesized by mixing *N*-methylimidazole (0.2 mol), *n*-butyl bromide (0.2 mol), and ethyl acetate (100 mL) in a microwave flask. The mixture was stirred at 85 °C under microwave irradiation (60 W) for 4 h (CEM Discover). The resulting pale-yellow liquid was washed with ethyl acetate and dried under a vacuum overnight. ^1^H NMR (DMSO-*d*_6_, 300 MHz) δ: 0.96 (t, 3H, *J* = 7.3), 1.39 (m, 2H), 1.92 (m, 2H), 4.14 (s, 3H), 4.36 (t, 2H, *J* = 7.3), 7.63 (s, 1H), 7.75 (s, 1H), 10.21 (s, 1H). Yield: 90%.

##### 1-Butyl-3-methylimidazolium Chloride (C_4_mimCl)

C_4_mimCl was obtained via ion exchange from C_4_mimBr using Amberlite IRN 78 resin (1.1 equiv. OH^–^·mL^–1^). The resin was first converted from the hydroxide (Resin–OH) to the chloride form (Resin-Cl) by treatment with aqueous HCl. A methanolic solution of C_4_mimBr (0.1 mol in 100 mL) was slowly passed through the Resin-Cl column, followed by rinsing with 50 mL methanol. The solvent was removed under reduced pressure, and the pale-yellow residue was dried under vacuum to yield pure C_4_mimCl. ^1^H NMR (DMSO-*d*_6_, 300 MHz) δ: 0.90 (t, 3H, *J* = 7.3), 1.26 (m, 2H), 1.77 (m, 2H), 3.87 (s, 3H), 4.19 (t, 2H, *J* = 7.3), 7.76 (s, 1H), 7.83 (s, 1H), 9.37 (s, 1H). Yield: 84%.

##### 1-Butyl-3-methylimidazolium Iodide (C_4_mimI)

C_4_mimI was synthesized similarly to C_4_mimBr, substituting *n*-butyl iodide for *n*-butyl bromide in the alkylation step. ^1^H NMR (DMSO-*d*_6_, 300 MHz) δ: 0.95 (t, 3H, *J* = 7.3), 1.36 (m, 2H), 1.85 (m, 2H), 4.05 (s, 3H), 4.28 (t, 2H, *J* = 7.3), 7.29 (s, 1H), 7.38 (s, 1H), 10.88 (s, 1H). Yield: 91%.

##### 1-Butyl-3-methylimidazolium Perchlorate (C_4_mimClO_4_)

C_4_mimClO_4_ was prepared by ion exchange from C_4_mimBr using Amberlite IRN 78 resin converted to the perchlorate form (Resin-ClO_4_) via treatment with aqueous perchloric acid (HClO_4_). A methanolic solution of C_4_mimBr (0.1 mol in 100 mL) was passed through the Resin-ClO_4_ column, followed by rinsing with 50 mL of methanol. The solvent was evaporated under reduced pressure, and the residue was dried under vacuum to afford the pure ionic liquid. ^1^H NMR (DMSO-*d*_6_, 300 MHz) δ: 0.92 (t, 3H, *J* = 7.3), 1.29 (m, 2H), 1.78 (m, 2H), 3.87 (s, 3H), 4.21 (t, 2H, *J* = 7.3), 7.75 (s, 1H), 7.85 (s, 1H), 9.32 (s, 1H). Yield: 82%.

##### 1-Ethyl-3-methylimidazolium Chloride (C_2_mimCl)

C_2_mimCl was synthesized by ion exchange from C_2_mimBr following the same procedure as that for C_4_mimCl. C_2_mimBr was prepared analogously to C_4_mimBr, replacing *n*-butyl bromide with ethyl bromide. ^1^H NMR C_2_mimCl (DMSO-*d*_6_, 300 MHz) δ: 1.32 (t, 3H, *J* = 7.3), 3.97 (s, 3H), 4.30 (qua, 2H, *J* = 7.3), 7.77 (s, 1H), 7.91 (s, 1H), 8.95 (s, 1H). Yield: 86%.

### Isothermal Titration Calorimetry (ITC) Measurements

An isothermal titration calorimeter (MicroCal VP-ITC, Malvern) was used to evaluate the enthalpy change of micellization and the CMC of SB3–14 in aqueous solutions containing varying concentrations of the imidazolium salts (100–1000 mmol L^–1^) at 298.15 ± 0.01 K. Prior to the calorimetric titrations, all solutions used in each experiment were degassed. In a typical experiment, the titrant, a 15 mmol·L^–1^ SB3–14 solution prepared in the same ionic liquid solution used as solvent, was injected into 1.4 mL of the corresponding ionic liquid solution placed in the calorimetric sample cell, where the mixture was stirred at 200 rpm. The reference cell was filled with deionized water, and an equilibration delay of 2000 s was applied before the first injection to ensure thermal stabilization. During all measurements, the reference power was maintained at 1 μW, with the feedback control disabled.

Each experiment consisted of at least 35 injections of 3–10 μL, spaced at intervals ranging from 300 to 1000 s, depending on the system studied. Injections were timed to ensure that the heat flow signal had returned to the baseline before the next addition. The resulting thermograms displayed well-defined thermal peaks corresponding to either heat release (exothermic) or absorption (endothermic), depending on the process occurring at each step. The area under each peak was integrated to determine the heat exchange, which was then normalized by the number of moles of SB3–14 added per injection to obtain the observed enthalpy change (Δ*H*_obs_). Data acquisition and analysis were performed using the software Origin, Version 2022 (OriginLab Corporation, Northampton, MA, USA), and for all obtained enthalpograms, the heat data from the first injection were discarded due to uncertainty in the actual injected volume.

## Results and Discussion

The experimental ITC enthalpograms are plotted in Figure 1 in terms of the observed enthalpy change, Δ*H*_obs_, as a function of the added surfactant concentration [SB3–14]. The enthalpograms were analyzed as before,^16^ defining the CMC as the inflection point of the sigmoidal micellization profile obtained from the ITC measurements in Figure 1. Fits of the ITC data in the regions of the sigmoidal profile below and above the CMC provided the observed enthalpy changes for monomeric (Δ*H*_obs_^mon^) and micellized (Δ*H*_obs_^mic^) surfactant and the standard enthalpy change for micellization was obtained as the difference between these two enthalpy changes at the CMC (eq 1)^32^

1 $${\Delta H}_{\mathrm{mic}}^{0}={\Delta H}_{\mathrm{obs}}^{\mathrm{mic}}-{\Delta H}_{\mathrm{obs}}^{\mathrm{mon}}$$

The standard Gibbs energies of micellization, Δ*G*_mic_^0^, were calculated from eq 2 ^6^

2 $${\Delta G}_{\mathrm{mic}}^{0}=\mathrm{RT}\ln\chi_{\mathrm{CMC}}=\mathrm{RT}\ln\left(\frac{\mathrm{CMC}}{55.5}\right)$$

where *R* is the molar gas constant, *T* is the absolute temperature, and χ_CMC_ = CMC/55.5 is the mole fraction of surfactant at the CMC (in mol L^–1^). The corresponding entropy changes of micellization (Δ*S*_mic_^0^) were then calculated from the relationship in eq 3

3 $${\Delta S}_{mic}^{0}=\frac{{\Delta H}_{mic}^{0}-{\Delta G}_{mic}^{0}}{T}$$

The thermodynamic parameters obtained from the ITC titration curves (CMC and Δ*H*_mic_^0^), along with those derived from them, were reproducible within ± 5%.

**Figure 1:**
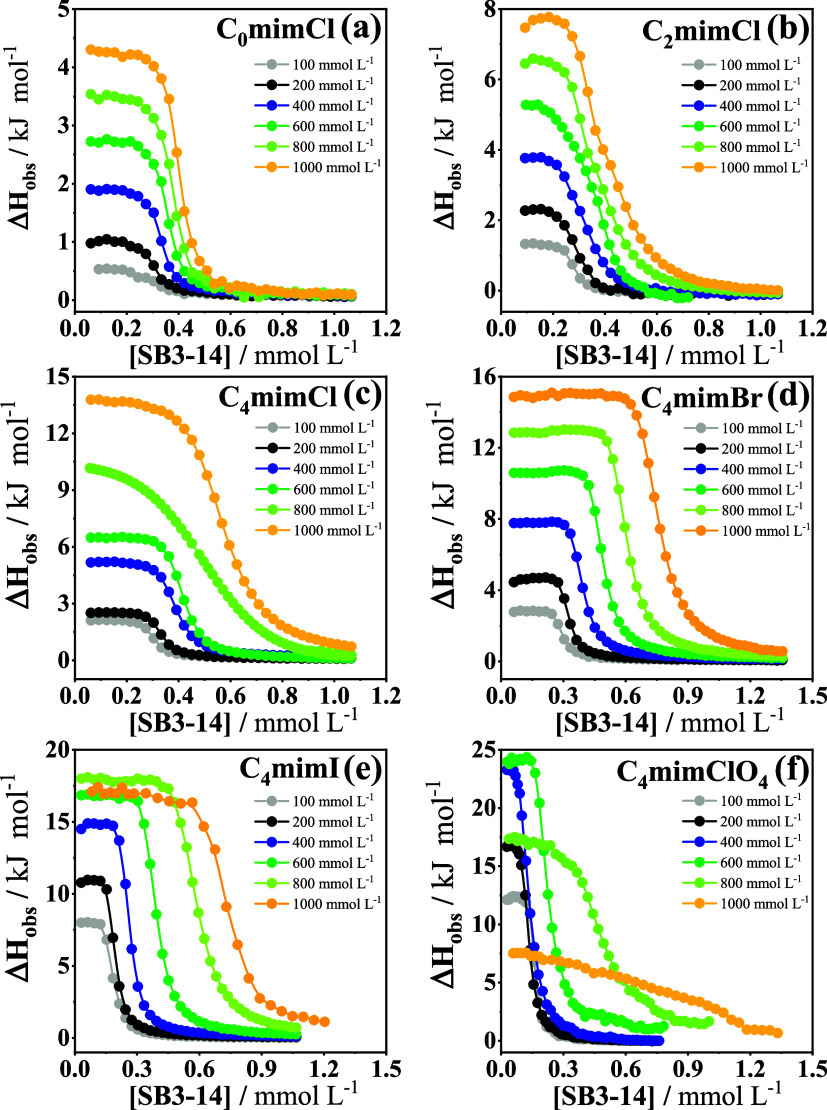
Observed enthalpy change (Δ*H*_obs_) versus SB3–14 concentration obtained by dilution of 15 mmol L^–1^ SB3–14 in an aqueous solution of the imidazolium salt (100–1000 mmol L^–1^) at 298.15 K: (a) C_o_mimCl, (b) C_2_mimCl, (c) C_4_mimCl, (d) C_4_mimBr, (e) C_4_mimI, and (f) C_4_mimClO_4_.

Figure 2 shows the variation of the CMC and the enthalpy of micellization of SB3–14 as a function of the concentration of added imidazolium salts (100–1000 mmol L^–1^). The corresponding numerical data are provided in Table S1 of the Supporting Information file. The CMC of SB3–14 in water in the absence of the imidazolium salts, determined by ITC at 298.15 K, is 0.27 ± 0.01 mmol L^–1^ and the enthalpy of micellization is only barely endothermic.^16^ As indicated in Figure 2a,b, four of the imidazolium salts, i.e., C_o_mimCl, C_2_mimCl, C_4_mimCl, and C_4_mimBr, increase the CMC of SB3–14 over the entire concentration range investigated (100–1000 mmol L^–1^), while the other two, C_4_mimI and C_4_mimClO_4_, initially decrease the CMC followed by an increase at much higher concentrations. An analogous behavior of an initial decrease followed by an increase in the CMC at much higher concentrations was reported for SB3–12 by Behera and Pandey upon addition of the imidazolium salt C_4_mimBF_4_,^24^ and, in a separate investigation,^25^ they compared the effects of C_4_mimBF_4_ and C_4_mimPF_6_ on SB3–12 micelles. These effects are distinct from those observed with inorganic salts such as NaCl, NaBr, NaI, and NaClO_4_, where the salt-induced decrease in the CMC follows the known relative affinities of the anions for the surface of SB3–14 micelles (Cl^–^ < Br^–^ < I^–^ < ClO_4_^–^)^6,8^ and is relatively insensitive to the nature or valence of the inorganic cation.^16^

**Figure 2:**
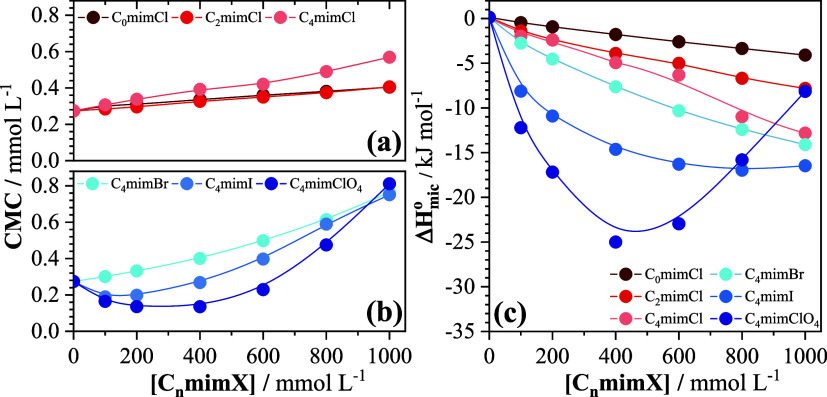
(a, b) CMC and (c) Δ*H*_mic_^0^ for the micellization of SB3–14 as a function of added imidazolium salt concentration at 298.15 K. Error bars are comparable to or smaller than the symbols for each data point. The curves are not actual data fits, but merely represent the trends of the data.

In contrast, Figure 2c shows that the enthalpies of micellization are consistently negative for all of the imidazolium salts, more similar to what was observed for inorganic salts.^16^ For the three chloride salts, the enthalpies become progressively more negative as the hydrophobicity of the imidazolium cation is increased from C_o_mimCl to C_2_mimCl to C_4_mimCl. For the same imidazolium cation, the enthalpies initially follow the sequence of affinities of the anion for the SB3–14 micelle-water interface, becoming more negative upon going from C_4_mimCl to C_4_mimBr to C_4_mimI to C_4_mimClO_4_. However, at higher concentrations of C_4_mimClO_4_, the enthalpy of micellization of SB3–14 becomes progressively less negative, approaching the value of C_2_mimCl at 1000 mmol L^–1^ (Figure 2c) and the ITC enthalpograms point to a reduction in the cooperativity of the micellization (broader and more poorly defined sigmoidals, respectively, for 800 and 1000 mmol L^–1^ added C_4_mimClO_4_ in Figure 1f). The corresponding variations of the Gibbs free energy of micellization and the contribution from the entropy of micellization (*T*Δ*S*_mic_^0^) at 298.15 K are plotted as a function of added imidazolium salt concentration in Figure 3 (the corresponding numerical data are provided in Table S1 of the Supporting Information file).

**Figure 3:**
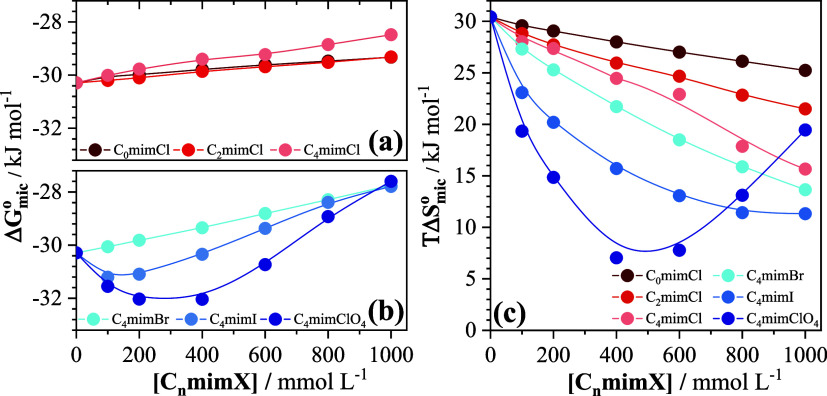
(a, b) Δ*G*_mic_^0^ and (c) *T*Δ*S*_mic_^0^ for the micellization of SB3–14 as a function of added imidazolium salt concentration at 298.15 K. Error bars are comparable to or smaller than the symbols for each data point. The curves are not actual data fits but merely represent the trends of the data.

In previous work,^16^ we demonstrated that the thermodynamics of the anion-headgroup (An-HG) interaction could be rationalized by eqs 4 and 5 for several simple zwitterionic surfactants in the presence of added sodium salts

4 $${\Delta H}_{\mathrm{mic}-\mathrm{An}}^{0}={\Delta H}_{\mathrm{mic}}^{0}+{\Delta H}_{\mathrm{An}-\mathrm{HG}}^{0}$$

5 $${T\Delta S}_{\mathrm{mic}-\mathrm{An}}^{0}={T\Delta S}_{\mathrm{mic}}^{0}+{T\Delta S}_{\mathrm{An}-\mathrm{HG}}^{0}$$

where Δ*H*_mic_^0^ and Δ*S*_mic_^0^ are the standard enthalpy and entropy changes for the micellization in water, and Δ*H*_mic‑An_^0^ and Δ*S*_mic‑An_^0^ are the corresponding values in the presence of the added sodium salt. The contributions of the anion-headgroup interaction to the enthalpy and entropy of micellization could then be estimated by rearranging eqs 4 and 5 to the form

6 $${\Delta H}_{\mathrm{An}-\mathrm{HG}}^{0}={\Delta H}_{\mathrm{mic}-\mathrm{An}}^{0}-{\Delta H}_{\mathrm{mic}}^{0}$$

7 $${\Delta S}_{\mathrm{An}-\mathrm{HG}}^{0}={\Delta S}_{\mathrm{mic}-\mathrm{An}}^{0}-{\Delta S}_{\mathrm{mic}}^{0}$$

The underlying assumption of eqs 6 and 7 is of course that the bulk of the effects due to differences in the chemical structure of the surfactants will cancel out by upon taking the difference between the enthalpies and entropies of micellization in the presence and absence of the added salt. The validity of this assumption was verified by demonstrating that values of Δ*H*_An‑HG_^0^ and Δ*S*_An‑HG_^0^ gave a single linear enthalpy–entropy compensation plot for three zwitterionic surfactants, i.e., SB3–14, its shorter-chain homologue SB3–12, and dodecyl phosphatidylcholine, which has the opposite orientation of the two headgroup charges.^16^ Moreover, because all of the added salts had the same counterion (Na^+^) and measurements on a series of inorganic chloride salts (NaCl, KCl, CsCl, LiCl, CaCl_2_, and AlCl_3_) demonstrated that both the CMC and the enthalpy of micellization of SB3–14 were insensitive to the nature or valence of the inorganic cation, explicit inclusion of an additional cation-dependent term was deemed to be unnecessary for the metal cation salts in our previous work.^16^

In contrast, the three imidazolium chloride salts C_o_mimCl, C_2_mimCl, and C_4_mimCl cause a net increase in the CMC of SB3–14 (Figure 2a,b) and cation-dependent changes in both the enthalpy and entropy of micellization of SB3–14 (Figures 2c and 3c), pointing to the necessity of explicit consideration of the effects of these organic cations. This is also evident in the enthalpy–entropy correlation for the imidazolium salts in Figure 4, which exhibits a significant deviation from the correlation observed in our previous work^16^ for sodium salts, with rather large dispersion of the data for C_4_mimI and C_4_mimClO_4_.

**Figure 4:**
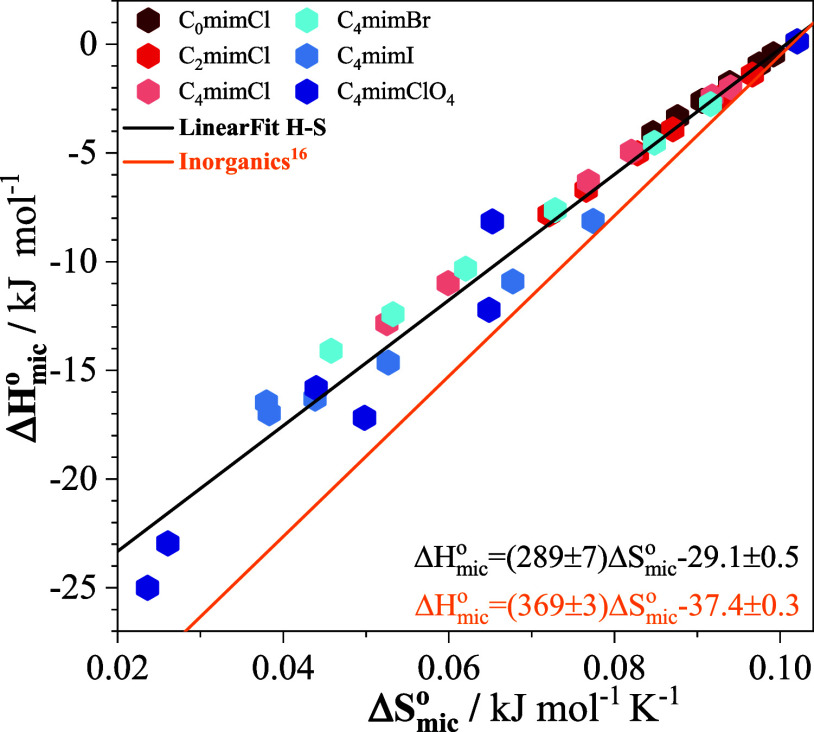
Enthalpy–entropy compensation plot for the micellization of SB3–14 in the presence of the imidazolium salts. The best-fit line (black), with a slope or compensation temperature of 289 ± 7 K, deviates significantly from the best-fit slope of 369 ± 3 K (indicated by the orange line) for the micellization of SB3–14 in the presence of the corresponding sodium salts.^16^

The simplest way to account for the imidazolium cation-zwitterionic interface interactions is to incorporate additional cation-dependent terms into eqs 4 and 5, respectively. In principle, these terms, Δ*H*_Cat_^0^ and *T*Δ*S*_Cat_^0^, will englobe not only interactions with the headgroup but also interactions with the hydrophobic portion of the surfactant as well.

8 $${\Delta H}_{mic-An}^{0}={\Delta H}_{mic}^{0}+{\Delta H}_{An-HG}^{0}+{\Delta H}_{Cat}^{0}$$

9 $${T\Delta S}_{\mathrm{mic}-\mathrm{An}}^{0}={T\Delta S}_{\mathrm{mic}}^{0}+{T\Delta S}_{\mathrm{An}-\mathrm{HG}}^{0}+{T\Delta S}_{\mathrm{Cat}}^{0}$$

There are two ways to gauge the adequacy of eqs 8 and 9 for describing the effects of the imidazolium salts. The first of these is to reference the data for the imidazolium salts C_4_mimBr, C_4_mimI, and C_4_mimClO_4_ to those of C_4_mimCl at the same concentrations and compare the results to the analogous data from our previous work^16^ for NaBr, NaI, and NaClO_4_ referenced against NaCl via eqs 10 and 11.

10 $${{\Delta \Delta H}_{\mathrm{mic}}^{0}=\Delta H}_{\mathrm{mic}-\mathrm{An}}^{0}-{\Delta H}_{\mathrm{mic}-\mathrm{Cl}}^{0}={\Delta H}_{\mathrm{An}-\mathrm{HG}}^{0}-{\Delta H}_{\mathrm{Cl}-\mathrm{HG}}^{0}$$

11 $${{\Delta \Delta S}_{\mathrm{mic}}^{0}=\Delta S}_{\mathrm{mic}-\mathrm{An}}^{0}-{\Delta S}_{\mathrm{mic}-\mathrm{Cl}}^{0}={\Delta S}_{\mathrm{An}-\mathrm{HG}}^{0}-{\Delta S}_{\mathrm{Cl}-\mathrm{HG}}^{0}$$

If this effectively cancels out the contributions from the cation, then the two sets of ΔΔ*H*_mic_^0^ and ΔΔ*S*_mic_^0^ data should fall onto a single enthalpy–entropy correlation plot, independent of the cation. As shown in Figure 5a, this is indeed the case, with a slope or compensation temperature of 350 ± 7 K, consistent with expectations (Figure 4) for an anion-dominated interaction term.

**Figure 5:**
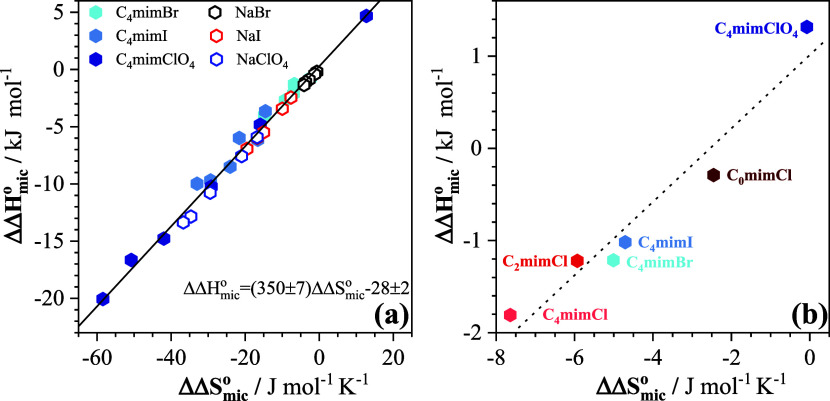
Enthalpy–entropy correlation plots based on (a) eqs 10 and 11 to factor out the contribution from the cations of the salt and (b) eqs 12 and 13 to factor out the contribution from the anion of the salt.

The second test is to examine whether the anion-dependent contributions cancel out when one references the data for the three different imidazolium chlorides C_0_mimCl, C_2_mimCl, and C_4_mimCl to those for NaCl at the same concentration of 100 mmol L^–1^, the maximum concentration employed in our previous work^16^ via eqs 12 and 13

12 ΔΔHmic−Cat0=ΔHmic−Im0−ΔHmic−Na0=ΔHIm0−ΔHNa0

13 ΔΔSmic−Cat0=ΔSmic−Im0−ΔSmic−Na0=ΔSIm0−ΔSNa0

Despite the limited number of data points and restricted numerical range of the data, the enthalpy–entropy correlation (Figure 5b) does exhibit a reasonably linear trend.

Additional information is provided by Figure 6a, in which the data of Figure 5, representing the contribution of the anions to the enthalpy and entropy of micellization, are plotted versus the concentration of added imidazolium salt. At the lower concentrations, the data follow the expected selectivity trend (ClO_4_^–^ > I^–^ > Br^–^) and both the enthalpy and entropy contributions become slightly more negative with increasing salt concentration. However, unlike the corresponding Na^+^ salts (Figure 6b), at higher concentrations, there are clear upward deviations indicative of a progressive weakening of the interaction of the anions with the zwitterionic interface, markedly so in the case of C_4_mimClO_4_. The onset of this upward trend shifts to lower concentration as the propensity of the anion for ion pairing with the C_4_mim^+^ cation increases.^18–22^ In the case of C_4_mimClO_4_, there is also a clear decrease in the cooperativity of the micellization of SB3–14 at 800 and 1000 mmol L^–1^ added salt. This behavior is mirrored in the seminal results of Behera and Pandey^24^ for SB3–12 in the presence of similarly high concentrations of C_4_mimBF_4_, which indicated a decrease in both the cooperativity of micellization and the apparent micellar aggregation number and changes in the interfacial properties as sensed by several fluorescence probes.

**Figure 6:**
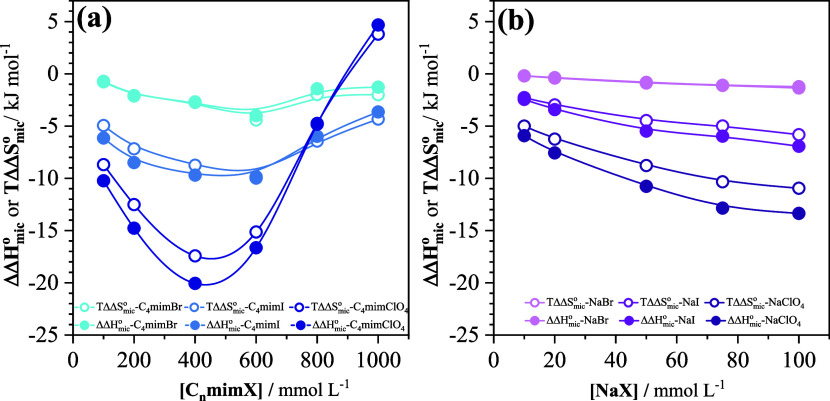
Contributions of the anion to the enthalpy and entropy of micellization of SB3–14 (from eqs 10 and 11, respectively) as a function of the concentration of (a) the imidazolium salts (this work) and (b) sodium salts (calculated from the data of ref 16).

## Conclusions

The picture that emerges from the present results is a competition between the organic cation and the anion for the zwitterionic interface, which favors the former for more weakly bound anions, such as chloride and bromide, especially at lower added salt concentrations. The predominance of ion pairing of the cation with the distal sulfonate groups of the surfactant leads to a decrease in the entropy of micellization that is only partially offset by the negative enthalpy contribution, resulting in the observed increase in the CMC. In contrast, for more strongly bound anions such as iodide and perchlorate, anion binding initially takes precedence over the interaction of the cation with the interface, leading to a decrease in the CMC. As the salt concentration is increased, competition from ion pairing of the imidazolium salt begins to weaken the anion binding to the zwitterionic interface. The observed behavior at very high concentrations of C_4_mimClO_4_ can be rationalized in terms of imidazolium salt-induced changes in the properties of the medium due to the onset of aggregate formation and microheterogeneity.

## Supplementary Material



## Data Availability

The structures of the three imidazolium cations and a comprehensive table of the CMCs and thermodynamic parameters determined in this work are provided in the Supporting Information.

## Notes

Chemical structures of the C_n_mim^+^ imidazolium cations, CMCs, and numerical values of the thermodynamic parameters determined in the present work (PDF)
