# Supplementary material for: Thermodynamics of the Micellization of a Simple Zwitterionic Surfactant in the Presence of Imidazolium Salts
Source: ACS Omega. 2025 Nov 4;10(45):54903–10. doi: 10.1021/acsomega.5c08858 (PMC12631678; doi:10.1021/acsomega.5c08858)
Supplement: Supplementary file 1 [file ao5c08858_si_001.pdf]

Supporting Information for:

Thermodynamics of the micellization of a simple zwitterionic surfactant in the presence of imidazolium salts.

Álvaro Javier Patiño-Agudelo,[1,2] Nicolas Keppeler,[1] Lucas Mendel de Oliveira Silva Martins[1] and Frank H. Quina*[1]

[1]*Departamento de Química Fundamental, Instituto de Química, Universidade de São Paulo, 05508-000, São Paulo, SP, Brazil.*

[2]*Departamento de Físico-Química, Instituto de Química, Universidade Estadual de Campinas, 13083-970, Campinas, SP, Brazil.*

E-mails:
patinoagudeloaj@gmail.com
nicolas.keppeler@usp.br
lucas.mendel@usp.br

**Corresponding author:* quina@usp.br

Scheme S1. Chemical structures of the C_nmim^+ imidazolium cations.

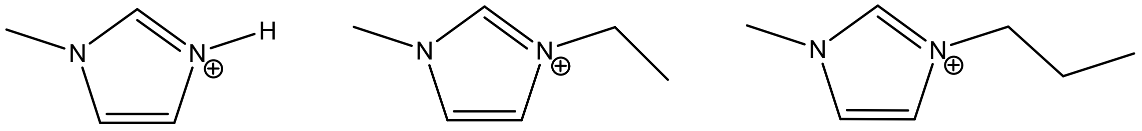

C_0mim^+ **C_2mim^+** **C_4mim^+**

Table 1S. Critical Micelle Concentration (CMC) and standard micellization parameters (ΔG^o_{mic}, ΔH^o_{mic}, and $T\Delta S^o_{mic}$) of SB3-14 in imidazolium salt solutions at 298.15 K.

Salt	[Salt]	CMC	ΔG^o_{mic}	ΔH^o_{mic}	$T\Delta S^o_{mic}$
**None**	0	0.27	-30.4	0.5	30.9
**C_0mimCl**	100	0.30	-30.0	-0.5	29.6
	200	0.31	-30.0	-0.9	29.1
	400	0.34	-29.8	-1.8	28.0
	600	0.36	-29.6	-2.6	27.0
	800	0.38	-29.5	-3.4	26.1
	1000	0.40	-29.3	-4.1	25.2
**C_2mimCl**	100	0.28	-30.2	-1.4	28.8
	200	0.30	-30.1	-2.4	27.7
	400	0.33	-29.9	-3.9	26.0
	600	0.35	-29.7	-5.0	24.7
	800	0.37	-29.5	-6.7	22.8
	1000	0.41	-29.3	-7.8	21.5
**C_4mimCl**	100	0.31	-30.0	-2.0	28.0
	200	0.34	-29.8	-2.4	27.4
	400	0.39	-29.4	-5.0	24.5
	600	0.42	-29.2	-6.3	22.9
	800	0.49	-28.9	-11.0	17.9
	1000	0.57	-28.5	-12.8	15.7
**C_4mimBr**	100	0.30	-30.1	-2.8	27.3
	200	0.33	-29.8	-4.5	25.3
	400	0.40	-29.4	-7.6	21.7
	600	0.50	-28.8	-10.3	18.5
	800	0.61	-28.3	-12.4	15.9
	1000	0.76	-27.8	-14.1	13.7
**C_4mimI**	100	0.19	-31.2	-8.1	23.1
	200	0.20	-31.1	-10.9	20.2
	400	0.27	-30.4	-14.6	15.7
	600	0.40	-29.4	-16.3	13.1
	800	0.59	-28.4	-17.0	11.4
	1000	0.75	-27.8	-16.5	11.3
**$C_4mimClO_4$**	100	0.17	-31.6	-12.2	19.3
	200	0.14	-32.0	-17.2	14.9
	400	0.14	-32.0	-25.0	7.0
	600	0.23	-30.7	-23.0	7.8
	800	0.48	-28.9	-15.8	13.1
	1000	0.81	-27.6	-8.2	19.5

The units of CMC and [salt] are mmol L^{-1} and the thermodynamic parameters are in kJ mol^{-1}.
